# Cognitive Improvements in Sinking Skin Flap Syndrome Using a Negative Pressure Helmet

**DOI:** 10.7759/cureus.98660

**Published:** 2025-12-07

**Authors:** Jian S Sabripour, Roberto A Martin, Anthony Pasarin, Regina Graham, Bryan Lieber

**Affiliations:** 1 Food Science and Human Nutrition, University of Florida, Gainesville, USA; 2 Osteopathic Medicine, Nova Southeastern University Dr. Kiran C. Patel College of Osteopathic Medicine, Davie, USA; 3 Surgery, Hospital Corporation of America (HCA) Florida Westside/Northwest Hospitals, Plantation, USA; 4 Allopathic Medicine, Nova Southeastern University, Davie, USA; 5 Neurological Surgery, Hospital Corporation of America (HCA) Florida Westside/Northwest Hospitals, Plantation, USA

**Keywords:** decompressive craniectomy (dc), intracranial pressure, midline shift, negative pressure helmet, sinking skin flap syndrome, syndrome of trephined

## Abstract

Sinking skin flap syndrome, also known as syndrome of the trephined, is a rare but serious complication that can occur following a decompressive craniectomy. It is characterized by neurological deficits that worsen with atmospheric pressure changes affecting the brain. Long-term treatment typically involves cranioplasty; however, temporizing measures are often needed when cranioplasty cannot be performed immediately. This case report introduces a minimally invasive approach using a negative-pressure helmet as a temporary therapeutic device aimed at prolonging survivability and restoring cognitive function in a patient with sinking skin flap syndrome.

In this case, the patient presented as a 71-year-old male with a prior craniotomy bone flap that became infected, requiring craniectomy, and developed aggressive syndrome of trephined shortly after surgery. A replacement skull flap was not readily available, and due to infection, placing foreign hardware was suboptimal, but the patient was experiencing significant and worsening mass effect, which warranted urgent intervention.

This case demonstrates the life-saving effects of using a negative pressure helmet as an emergency measure to relieve midline shift caused by sinking skin flap syndrome. This method, while novel, may serve to be integrated as a widely used treatment for certain patients with sinking skin flap syndrome.

## Introduction

Sinking skin flap syndrome (SSFS) is a common and potentially fatal complication observed in patients who have undergone decompressive craniectomy (DC), often due to traumatic brain injury or stroke [1]. The syndrome arises when atmospheric pressure causes the brain to sink at the site of the craniectomy, leading to mass effect and neurologic deficits [2]. Estimates of its prevalence vary, with rates between 13% and 53% reported in large DCs [3]. The definitive treatment for SSFS is cranioplasty; however, some patients are not immediate candidates due to medical instability or other contraindications, such as infection [4]. This report describes a case of acute SSFS successfully managed with an improvised negative pressure helmet (NegPresHelm), which served as a non-invasive bridge to cranioplasty in a patient who could not undergo immediate surgical reconstruction.

## Case presentation

**Figure 1:**
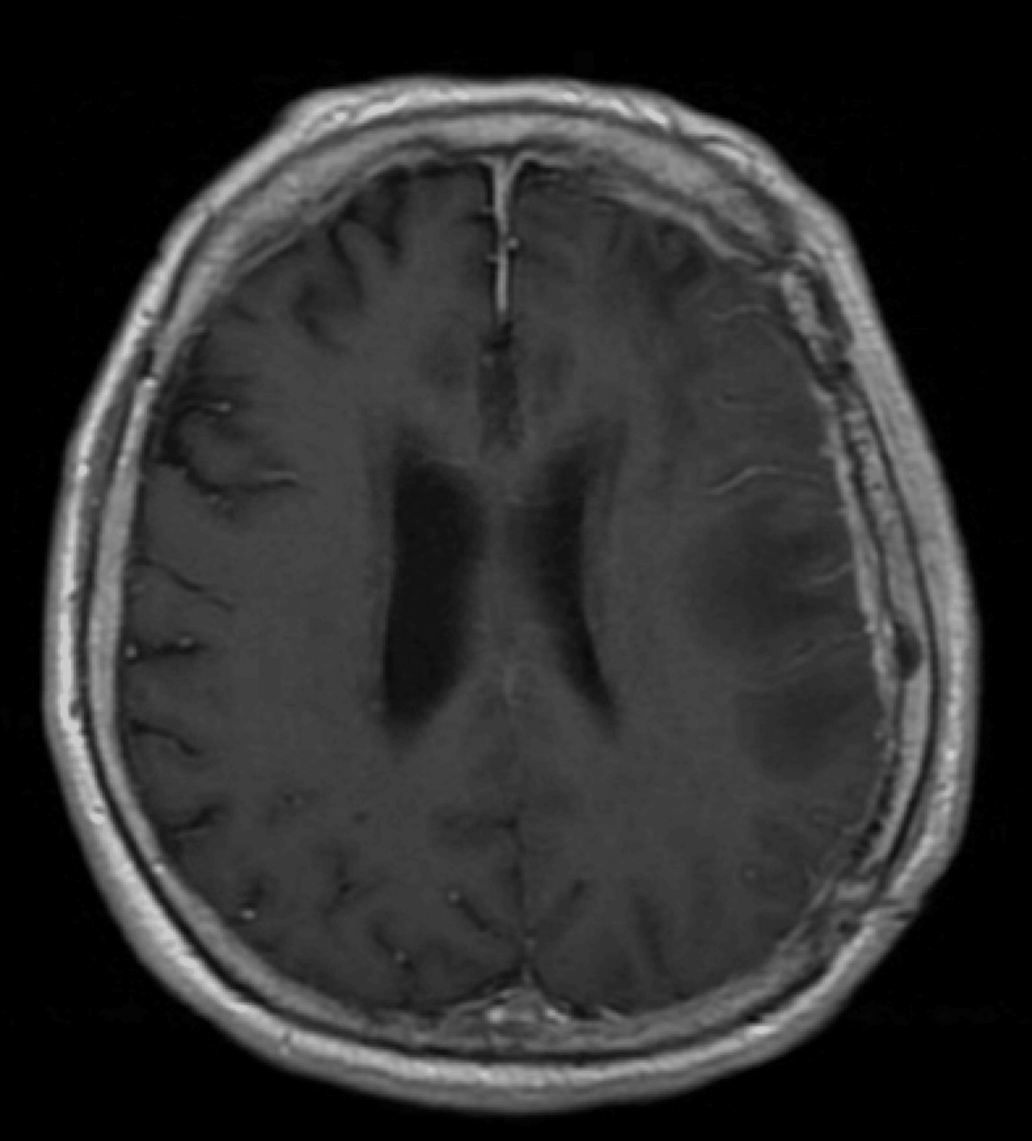
Initial Presentation MRI of the brain displays pachymeningeal enhancement with underlying parenchymal edema, prompting immediate surgery.

A 71-year-old cisgender male with poorly controlled diabetes mellitus and a history of traumatic left subdural hematoma underwent a prior craniotomy one year earlier. He presented with worsening aphasia and purulent crusting over his craniotomy site. MRI of the brain showed pachymeningeal enhancement with underlying parenchymal edema (Figure 1) consistent with subdural empyema and cerebritis, as well as eroded hardware. The patient subsequently underwent emergent left hemicraniectomy with debridement of infected tissue. Intraoperatively, the patient was found to have epidural involvement of the inner skull table, making the reuse of the autologous bone flap for future cranioplasty unsuitable.

**Figure 2:**
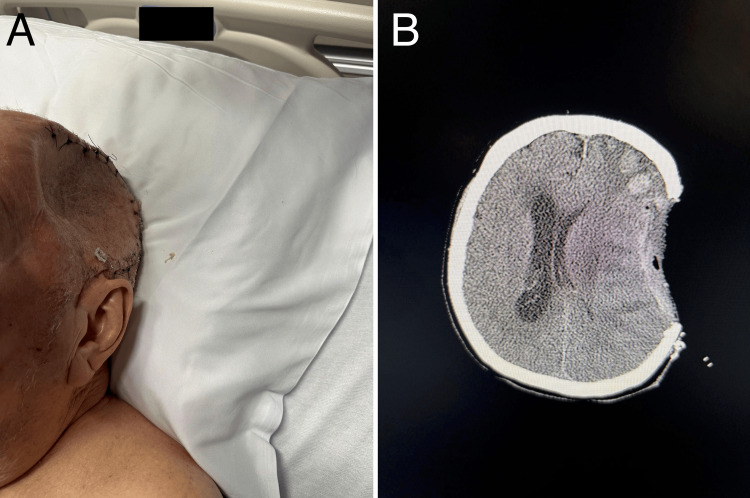
Acute Sinking Skin Flap Syndrome Patient exhibits cranial depression consistent with Sinking Skin Flap Syndrome (SSFS) at the craniectomy site (A). Non-contrast CT imaging depicts a 9mm left-to-right midline shift (B).

Postoperatively, the patient initially demonstrated improved mental status. However, by postoperative day (POD) four, he developed worsening confusion, aphasia, and new-onset right-sided hemiparesis. Physical examination revealed a prominent depression at the site of the craniectomy (Figure 2A), and non-contrast CT of the head confirmed a left-to-right midline shift of 9 mm (Figure 2B). This worsened to 15 mm over the following days, consistent with SSFS.

Due to active infection and delays in manufacturing a custom implant, cranioplasty was not immediately feasible. In an effort to prevent progression to subfalcine herniation, a novel non-invasive intervention was devised in the form of a negative pressure helmet (NegPresHelm), which could shield the patient’s cranial vault from the effects of atmospheric pressure. The helmet was designed to create a localized negative pressure environment around the craniectomy site, counteracting the atmospheric pressure and helping to pull the brain back into place.

**Figure 3:**
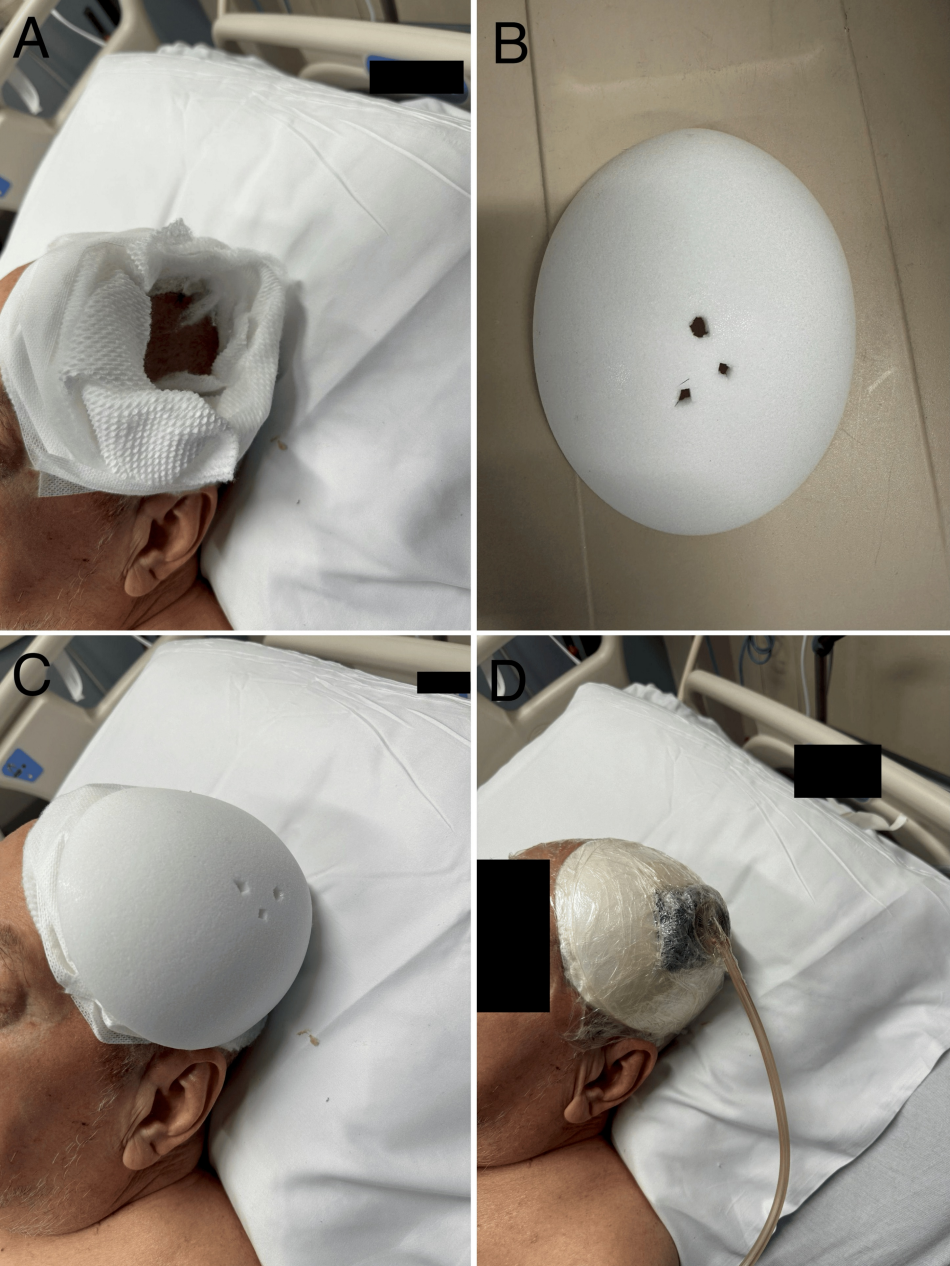
Negative Pressure Helmet Soft cast paddings and foam (A), as well as a Su-Por® cranial implant with perforations (B) carefully placed over the craniectomy site (C) and sealed by Tegaderm film dressings to create an air-tight space allowing suction from a wound vacuum to reach the cranial defect (D).

The helmet was created using a Poriferous Su-Por® (Poriferous, LLC, in Newnan, Georgia) sample Cranial Dome implant made from pure high-density polyethylene (HDPE), and a standard wound vacuum. A cushioned layer was made surrounding the craniectomy site using soft cast padding and foam (Figure 3A). The helmet was perforated and placed on top of the cushion so that no contact would be made with the patient’s skin to ensure patient comfort (Figure 3B, 3C). Tegaderm film dressings were placed on top of and surrounding the helmet, creating an airtight space with a hole for placement of the wound vacuum. The wound vacuum was then set to 25 mmHg, and the pressure seal was confirmed (Figure 3D).

**Figure 4:**
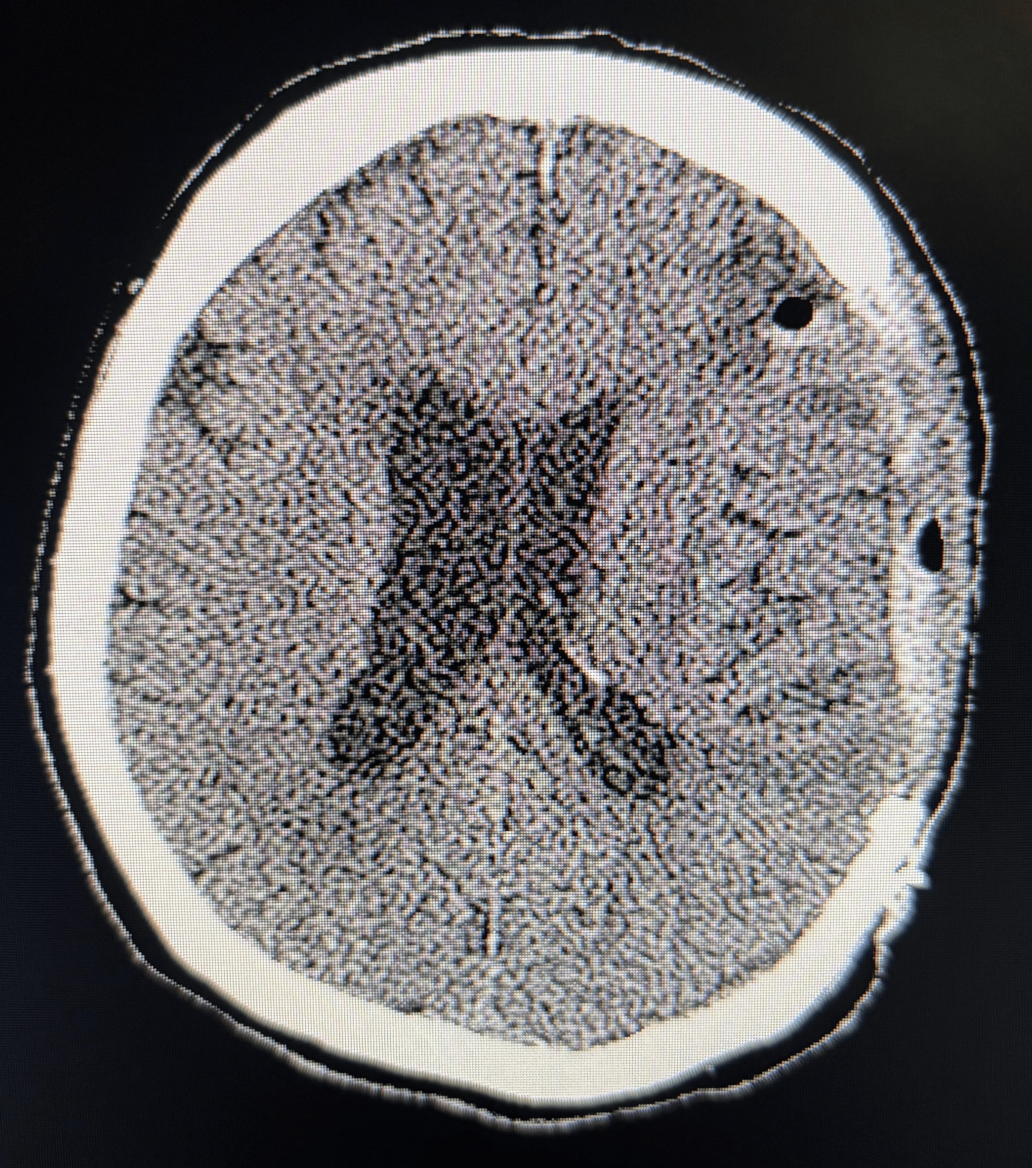
Resolution of Midline Shift CT imaging shows complete resolution of midline shift after NegPresHelm intervention.

On POD eight, the NegPresHelm was applied. Within 24 hours, the patient exhibited marked clinical improvement: enhanced alertness, improved speech fluency, and regained motor strength in the right extremities. Repeat CT imaging showed a reduction of the midline shift to 9 mm. On POD10, the vacuum pressure was increased to -50 mmHg, resulting in further radiographic and clinical improvement. (Figure 4).

**Figure 5:**
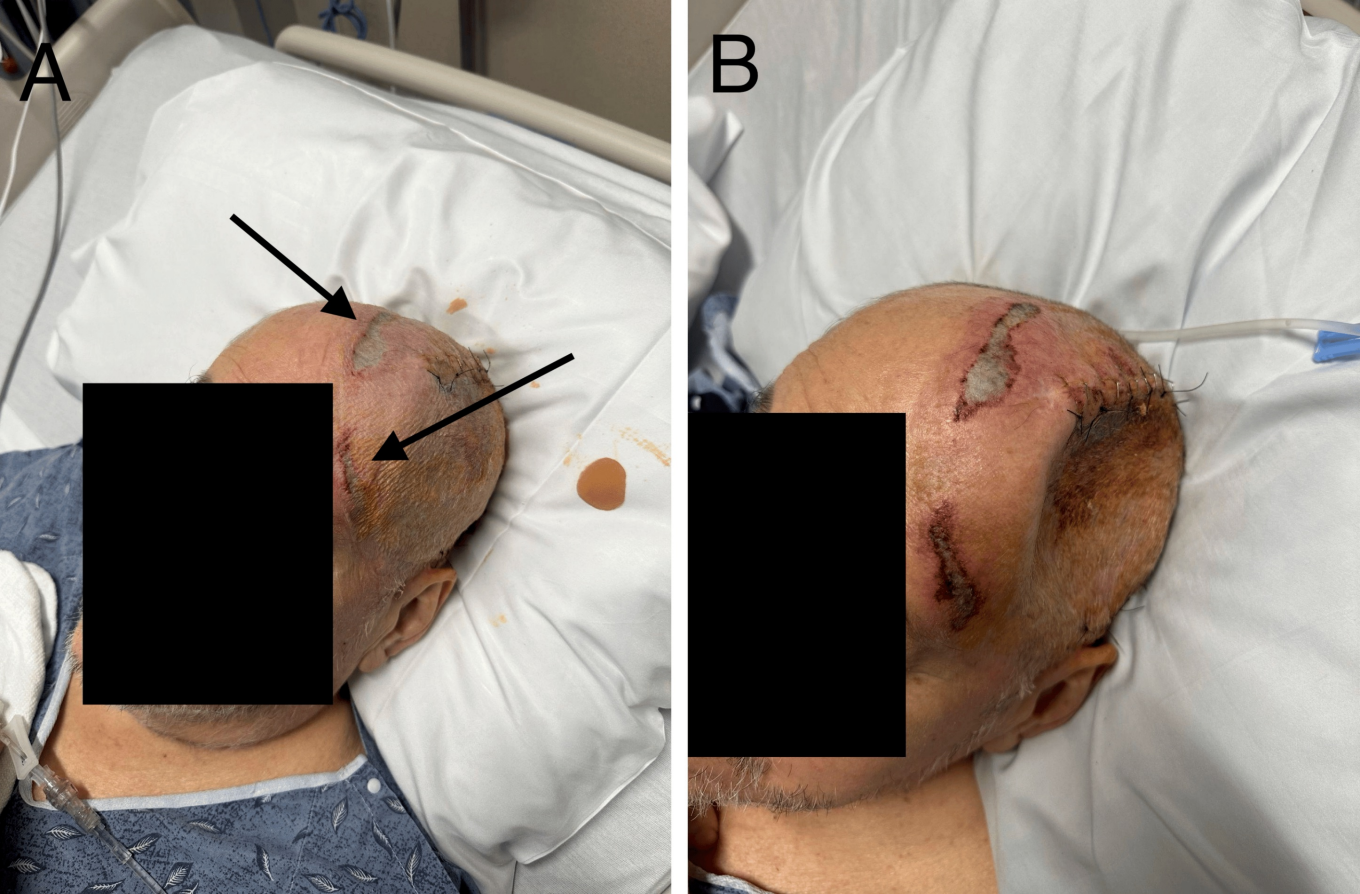
Cranial Shape Resolution, Side Effects, and Recurrence of Sunken Skin Flap Upon NegPresHelm removal, cranial shape is resolved, and circumferential pressure ulcer formation is identified at the craniectomy site (A). One day post- NegPresHelm removal, cranial depression is observed (B).

By POD 14, the patient's midline shift had resolved, and the helmet was removed; however, pressure ulcers were discovered circumferentially overlying the border of the patient’s craniectomy defect (Figure 5A). The depth of these pressure ulcers was unknown at the time, so to prevent tissue necrosis and suboptimal wound healing after cranioplasty, the NegPresHelm was discontinued, and the patient was placed in the Trendelenburg position in an attempt to stave off the recurrence of SSFS. The following day, however, the patient’s cognitive status deteriorated again, with a recurrent sunken flap and CT imaging revealing an 8 mm midline shift (Figure 5B). The patient's condition continued to decline until definitive cranioplasty was performed on POD21.

At two-week follow-up, the patient demonstrated notable neurologic progress, with normalization of his ICP and resolution of his expressive aphasia and conceptual apraxia. However, he had not regained the full cognitive function observed during NegPresHelm use. He demonstrated confabulation and short-term memory deficits, and persistent right upper extremity stiffness. CT imaging showed that the brain had not completely re-expanded to fill the cranioplasty volume.

**Figure 6:**
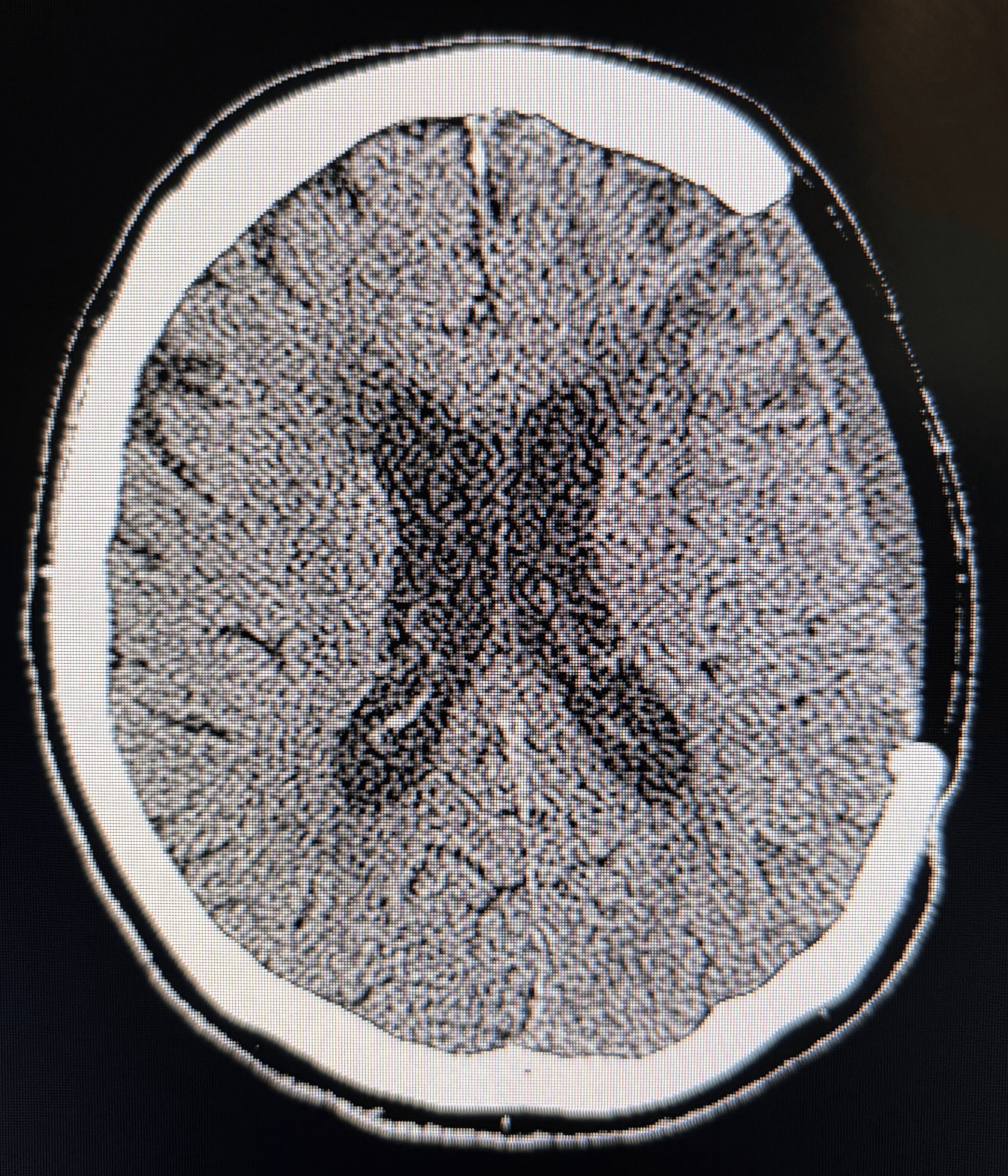
Long-term Resolution 5 months post-cranioplasty, CT imaging shows significant improvement in the patient’s mass effect.

At five-month follow-up, the patient had near-complete resolution of the right arm spasticity and marked cognitive recovery, with normalization of midline structures on CT imaging (Figure 6).

## Discussion

This case highlights the potential of a negative pressure helmet (NegPresHelm) as a novel, non-invasive, and innovative temporary measure for the management of acute SSFS. The mechanism of action likely involves the restoration of normal intracranial pressure dynamics by reducing the atmospheric pressure burden over the craniectomy site, thereby mitigating midline shift and associated neurological deficits [5].

Craniectomies are generally performed to decompress the brain, often as a result of major cerebral edema, and the typical onset of SSFS occurs between 28 and 188 days postoperatively [3,6]. In this case, however, SSFS developed as early as POD4 and the patient had an infected skull flap, prompting immediate removal. Acute onset of SFSS could be due to the absence of cerebral edema, and the removal of tack-up sutures from the patient’s original craniotomy may have also contributed by creating space between the brain and skin.

Notably, the acute nature of this case may be a key factor in its success. Chronic SSFS may lead to the development of sclerotic skin at the craniectomy site. As a result, negative pressure helmets may be ineffective due to the formation of a barrier from the sclerotic skin, which impedes the brain’s ability to shift. Watchi et al. in 2022 report a case in which a NegPresHelm failed to produce improvement in a chronic SSFS patient, likely due to a fibrotic barrier [5]. This underscores the value of early intervention. The absence of sclerotic changes in the skin and subcutaneous tissue likely facilitated the observed reversal of midline shift and neurological improvements. Conversely, once fibrosis develops, tissue rigidity may preclude re-expansion of the underlying brain parenchyma even with external mechanical support.

Importantly, the materials used in this NegPresHelm assembly are widely available in most hospital settings. Standard wound care supplies such as Tegaderm dressings, soft cast padding, and a wound vacuum device were repurposed to create a sealed system. The cranial contour was achieved using a sample cranial implant, though this could be replaced with any firm, dome-shaped object that can fit over the craniectomy site. In a similar case, reported by Courville et al., constructed a custom helmet using plaster of Paris and 4x4 gauze, further supporting the accessibility and adaptability of this approach [7].

Due to the limited literature for comparable cases, the choice of negative pressure levels was empirical, guided by the goal of applying sufficient counterforce to alleviate midline shift while minimizing the risk of cerebrospinal fluid aspiration. -25 mmHg was first chosen and increased to -50 mmHg based on the patient's clinical and radiographic response. The literature describes pressures as high as -75 mmHg being necessary for midline shift correction [5,7]. However, at -50 mmHg, the mass effect had corrected, thus there was no need to increase pressure. In fact, it is possible that decreasing the pressure once the midline shift is resolved could have prevented or slowed the formation of pressure ulcers.

The primary complication encountered was superficial skin ulceration along the helmet’s contact margins. This was attributed to prolonged contact pressure and potentially excessive suction. These findings highlight the need for refinement in the NegPresHelm design, specifically, the development of translucent, form-fitting helmets with regulated pressure settings and built-in pressure distribution systems to mitigate the risk of pressure ulcers and necrosis.

Because of the novel nature of this intervention, informed consent was obtained from the patient’s legal representative, and the intervention was approved by institutional oversight, given the lack of viable alternatives. However, widespread implementation of similar strategies will require formal investigation to validate safety and efficacy.

## Conclusions

In this case, a negative pressure helmet reversed neurologic decline and midline shift associated with acute SSFS in a patient awaiting cranioplasty. In situations where surgery is delayed, this may serve as a temporary non-invasive, low-cost intervention. Further research and clinical trials are warranted to establish standardized protocols and evaluate long-term efficacy, safety, and comfort.
